# Supplementary material for: The Impact of Covid-19 and the Effect of Psychological Factors on Training Conditions of Handball Players
Source: Int J Environ Res Public Health. 2020 Sep 5;17(18):6471. doi: 10.3390/ijerph17186471 (PMC7558666; doi:10.3390/ijerph17186471)
Supplement: Supplementary file 1 [file ijerph-17-06471-s001.pdf]

Balonmano y Covid-19

El presente cuestionario tiene como objetivo recopilar información acerca de la salud y el rendimiento de jugadores/ras de balonmano durante el periodo extraordinario de restricción de la actividad habitual con motivo del COVID-19. Diferentes investigadores de varias universidades españolas colaboran en este estudio para valorar cómo afecta el periodo de aislamiento a los hábitos de jugadores de balonmano en España.

La información que usted proporcione tendrá fines de investigación, es anónima, y será tratada con estricta confidencialidad, de acuerdo a la Ley Orgánica de Protección de Datos - 3/2018. Participar en esta encuesta no implica compromiso alguno y es totalmente voluntario. Usted solamente debería participar si tiene voluntad de hacerlo. Puede abandonar la encuesta en el momento en el que estime oportuno.

La duración aproximada del cuestionario es de 6-7 minutos.

SI DA SU CONSENTIMIENTO PULSE SIGUIENTE

Si quiere plantear alguna duda acerca del estudio o del cuestionario puede hacerlo a través del siguiente e-mail: alfonso.delarubia@upm.es

* Required

Cuestiones demográficas

Instrucciones: A continuación, encontrará algunas cuestiones sobre sus caracteristicas demográficas.

1. Sexo *

Mark only one oval.

- ( ) Hombre
- ( ) Mujer
- ( ) Otros

2. Edad *

Mark only one oval.

- 11
- 12
- 13
- 14
- 15
- 16
- 17
- 18
- 19
- 20
- 21
- 22
- 23
- 24
- 25
- 26
- 27
- 28
- 29
- 30
- 31
- 32
- 33
- 34
- 35
- 36
- 37
- 38
- 39
- 40 o más

3. ¿Cuál es tu nivel de estudios? *

Mark only one oval.

- Fue menos de 5 años a la escuela
- Educación primaria completa o fue a la escuela al menos 5 años
- ESO, EGB y Bachiller Elemental; Certificados de Estudios Primarios y de Escolaridad
- Bachiller, BUP, COU, Bachiller Superior, PREU y similares
- Ciclos Formativos de Grado Medio, FPI, Gr. Medio/FP básica, y similares
- Ciclos Formativos de Grado Superior, FPII y equivalentes
- Estudios universitarios
- Máster o Doctorado

4. Nacionalidad *

Mark only one oval.

- Afganistán
- Albania
- Alemania
- Andorra
- Angola
- Antigua y Barbuda
- Arabia Saudita
- Argelia
- Argentina
- Armenia
- Australia
- Austria
- Azerbaiyán
- Bahamas
- Bangladés
- Barbados
- Baréin
- Bélgica
- Belice
- Benín
- Bielorrusia
- Birmania/Myanmar
- Bolivia
- Bosnia y Herzegovina
- Botsuana
- Brasil
- Brunéi
- Bulgaria
- Burkina Faso
- Burundi
- Bután
- Cabo Verde
- Camboya
- Camerún
- Canadá
- Catar
- Chad

- Chile
- China
- Chipre
- Ciudad del Vaticano
- Colombia
- Comoras
- Corea del Norte
- Corea del Sur
- Costa de Marfil
- Costa Rica
- Croacia
- Cuba
- Dinamarca
- Dominica
- Ecuador
- Egipto
- El Salvador
- Emiratos Árabes Unidos
- Eritrea
- Eslovaquia
- Eslovenia
- España
- Estados Unidos
- Estonia
- Etiopía
- Filipinas
- Finlandia
- Fiyi
- Francia
- Gabón
- Gambia
- Georgia
- Ghana
- Granada
- Grecia
- Guatemala
- Guyana
- Guinea
- Guinea ecuatorial
- Guinea-Bisáu

- Haití
- Honduras
- Hungría
- India
- Indonesia
- Irak
- Irán
- Irlanda
- Islandia
- Islas Marshall
- Islas Salomón
- Israel
- Italia
- Jamaica
- Japón
- Jordania
- Kazajistán
- Kenia
- Kirguistán
- Kiribati
- Kuwait
- Laos
- Lesoto
- Letonia
- Líbano
- Liberia
- Libia
- Liechtenstein
- Lituania
- Luxemburgo
- Macedonia del Norte
- Madagascar
- Malasia
- Malaui
- Maldivas
- Malí
- Malta
- Marruecos
- Mauricio
- Mauritania

- México
- Micronesia
- Moldavia
- Mónaco
- Mongolia
- Montenegro
- Mozambique
- Namibia
- Nauru
- Nepal
- Nicaragua
- Níger
- Nigeria
- Noruega
- Nueva Zelanda
- Omán
- Países Bajos
- Pakistán
- Palaos
- Panamá
- Papúa Nueva Guinea
- Paraguay
- Perú
- Polonia
- Portugal
- Reino Unido
- República Centroafricana
- República Checa
- República del Congo
- República Democrática del Congo
- República Dominicana
- República Sudafricana
- Ruanda
- Rumanía
- Rusia
- Samoa
- San Cristóbal y Nieves
- San Marino
- San Vicente y las Granadinas
- Santa Lucía

- Santo Tomé y Príncipe
- Senegal
- Serbia
- Seychelles
- Sierra Leona
- Singapur
- Siria
- Somalia
- Sri Lanka
- Suazilandia
- Sudán
- Sudán del Sur
- Suecia
- Suiza
- Surinam
- Tailandia
- Tanzania
- Tayikistán
- Timor Oriental
- Togo
- Tonga
- Trinidad y Tobago
- Túnez
- Turkmenistán
- Turquía
- Tuvalu
- Ucrania
- Uganda
- Uruguay
- Uzbekistán
- Vanuatu
- Venezuela
- Vietnam
- Yemen
- Yibuti
- Zambia
- Zimbabue

5. ¿Donde estas residiendo actualmente? *

Mark only one oval.

- Afganistán
- Albania
- Alemania
- Andorra
- Angola
- Antigua y Barbuda
- Arabia Saudita
- Argelia
- Argentina
- Armenia
- Australia
- Austria
- Azerbaiyán
- Bahamas
- Bangladés
- Barbados
- Baréin
- Bélgica
- Belice
- Benín
- Bielorrusia
- Birmania/Myanmar
- Bolivia
- Bosnia y Herzegovina
- Botsuana
- Brasil
- Brunéi
- Bulgaria
- Burkina Faso
- Burundi
- Bután
- Cabo Verde
- Camboya
- Camerún
- Canadá
- Catar
- Chad

- Chile
- China
- Chipre
- Ciudad del Vaticano
- Colombia
- Comoras
- Corea del Norte
- Corea del Sur
- Costa de Marfil
- Costa Rica
- Croacia
- Cuba
- Dinamarca
- Dominica
- Ecuador
- Egipto
- El Salvador
- Emiratos Árabes Unidos
- Eritrea
- Eslovaquia
- Eslovenia
- España
- Estados Unidos
- Estonia
- Etiopía
- Filipinas
- Finlandia
- Fiyi
- Francia
- Gabón
- Gambia
- Georgia
- Ghana
- Granada
- Grecia
- Guatemala
- Guyana
- Guinea
- Guinea ecuatorial
- Guinea-Bisáu

- Haití
- Honduras
- Hungría
- India
- Indonesia
- Irak
- Irán
- Irlanda
- Islandia
- Islas Marshall
- Islas Salomón
- Israel
- Italia
- Jamaica
- Japón
- Jordania
- Kazajistán
- Kenia
- Kirguistán
- Kiribati
- Kuwait
- Laos
- Lesoto
- Letonia
- Líbano
- Liberia
- Libia
- Liechtenstein
- Lituania
- Luxemburgo
- Macedonia del Norte
- Madagascar
- Malasia
- Malaui
- Maldivas
- Malí
- Malta
- Marruecos
- Mauricio
- Mauritania

- México
- Micronesia
- Moldavia
- Mónaco
- Mongolia
- Montenegro
- Mozambique
- Namibia
- Nauru
- Nepal
- Nicaragua
- Níger
- Nigeria
- Noruega
- Nueva Zelanda
- Omán
- Países Bajos
- Pakistán
- Palaos
- Panamá
- Papúa Nueva Guinea
- Paraguay
- Perú
- Polonia
- Portugal
- Reino Unido
- República Centroafricana
- República Checa
- República del Congo
- República Democrática del Congo
- República Dominicana
- República Sudafricana
- Ruanda
- Rumanía
- Rusia
- Samoa
- San Cristóbal y Nieves
- San Marino
- San Vicente y las Granadinas
- Santa Lucía

- Santo Tomé y Príncipe
- Senegal
- Serbia
- Seychelles
- Sierra Leona
- Singapur
- Siria
- Somalia
- Sri Lanka
- Suazilandia
- Sudán
- Sudán del Sur
- Suecia
- Suiza
- Surinam
- Tailandia
- Tanzania
- Tayikistán
- Timor Oriental
- Togo
- Tonga
- Trinidad y Tobago
- Túnez
- Turkmenistán
- Turquía
- Tuvalu
- Ucrania
- Uganda
- Uruguay
- Uzbekistán
- Vanuatu
- Venezuela
- Vietnam
- Yemen
- Yibuti
- Zambia
- Zimbabue

6. ¿En que comunidad autónoma reside usted? *

Mark only one oval.

- Ninguna
- Andalucía
- Aragón
- Asturias
- Cantabria
- Castilla la Mancha
- Castilla León
- Cataluña
- Ceuta *
- Extremadura
- Galicia
- Islas Baleares
- Islas Canarias
- la Rioja
- Madrid
- Melilla *
- Murcia
- Navarra
- País Vasco
- Valencia

7. Categoria *

Mark only one oval.

- Asobal (division de honor masculina)
- División de honor plata masculina
- Primera nacional masculina
- Segunda nacional masculina o inferiores
- Liga guerreras Iberdrola (division de honor femenina)
- Division de honor plata femenina
- Segunda nacional fenenina o inferiores

8. ¿Has sido convocado por tu selección nacional o autonómica en las dos últimas temporadas? *

Mark only one oval per row.

	Si	No
Autonómica	◯	◯
Nacional	◯	◯

9. Puesto *

Mark only one oval.

- ◯ Portero/a
- ◯ Extremo
- ◯ Lateral
- ◯ Central
- ◯ Pivote

10. ¿Con cuantas personas estas conviviendo durante el confinamiento? *

Mark only one oval.

- ◯ Estoy solo
- ◯ 1
- ◯ 2
- ◯ 3
- ◯ 4
- ◯ 5
- ◯ 6 o más

11. ¿Cómo de grande es la vivienda en la que está realizando el confinamiento en metros cuadrados? *

Mark only one oval.

- ◯ Menos de 30 metros cuadrados
- ◯ Entre 31 y 50 metros cuadrados
- ◯ Entre 51 y 70 metros cuadrados
- ◯ Entre 71 y 90 metros cuadrados
- ◯ Entre 91 y 110 metros cuadrados
- ◯ Mas de 111 metros cuadrados

12. ¿De qué espacios dispone para realizar actividad en su vivienda? *

Check all that apply.

- [ ] Salón y/o habitación tamaño medio para entrenar
- [ ] Terraza con espacio para moverme
- [ ] Jardín con espacio para poder correr 15 m
- [ ] Garaje libre de espacios
- [ ] Otros

13. ¿De qué material para entrenar dispone para realizar actividad en su vivienda? *

Check all that apply.

- [ ] Máquinas cardiovasculares (bici estática, elíptica, cinta)
- [ ] Colchoneta o similar
- [ ] Materiales para sobrecarga (discos, barras, mancuernas, kettlebell, balones medicinales)
- [ ] Materiales suspensión o similar (TRX)
- [ ] Materiales inestables (bosu-fitball-togu)
- [ ] Materiales elásticos (Bandas elásticas)
- [ ] Materiales técnicos (balones, porterías)
- [ ] Materiales coordinación y agilidad (escaleras, aros, conos, combas, picas)
- [ ] Máquinas de gimnasio
- [ ] No tengo material

14. ¿Te encuentras lesionad@ durante el confinamiento? *

Mark only one oval.

- ( ) Si
- ( ) No

15. Escala de esfuerzo percibido media (RPE) de una semana de entrenamiento [0-10] *

Mark only one oval per row.

	Nada	1	2	3	4	5	6	7	8
Previo al confinamiento	( )	( )	( )	( )	( )	( )	( )	( )	( )
Durante el confinamiento	( )	( )	( )	( )	( )	( )	( )	( )	( )

16. ¿Cuantas días de entrenamiento realiza / realizaba semanalmente incluyendo el partido? *

Mark only one oval per row.

	Uno	Dos	Tres	Cuatro	Cinco	Seis	Siete
Previo al confinamiento							
Durante el confinamiento							

17. ¿Cuantas horas de entrenamientos semanales realizaba previo al confinamiento incluyendo el partido? *

Mark only one oval.

- 0
- 1
- 2
- 3
- 4
- 5
- 6
- 7
- 8
- 9
- 10
- 11
- 12
- 13
- 14
- 15
- 16
- 17
- 18
- 19
- 20
- 21
- 22
- 23
- 24
- 25
- 26
- 27
- 28
- 29
- 30 o más

18. ¿Cuantas horas de entrenamientos semanales esta realizando durante el confinamiento? *

Mark only one oval.

- 0
- 1
- 2
- 3
- 4
- 5
- 6
- 7
- 8
- 9
- 10
- 11
- 12
- 13
- 14
- 15
- 16
- 17
- 18
- 19
- 20
- 21
- 22
- 23
- 24
- 25
- 26
- 27
- 28
- 29
- 30 o más

19. ¿Qué relación tiene o ha tenido usted con el coronavirus? *

Mark only one oval.

- Ninguna
- Sí, la padezco actualmente
- Sí, me he curado
- Lo desconozco

20. ¿Tiene o ha tenido Usted personas en su entorno que padezcan o hayan padecido el coronavirus? Marque tantas respuestas como considere oportunas *

Check all that apply.

- [ ] Ninguna
- [ ] Sí, conocidos
- [ ] Sí, amigos
- [ ] Sí, familiares
- [ ] Sí, compañeros de equipo /cuerpo técnico

21. ¿Ha sufrido algún Expediente Temporal de Regulación de Empleo (ERTE), suspensión de pagos o reducción del salario durante este periodo de confinamiento? *

Mark only one oval.

- Si
- No

22. ¿Indique su grado de preocupación con respecto al la posibilidad de sufrir un Expediente Temporal de Regulación de Empleo (ERTE), suspensión de pagos o reducción del salario durante este periodo de confinamiento? *

Mark only one oval.

	1	2	3	4	5	6	7	8	9	10	
Nada											Máximo

23. En relación al sueño ¿Cuantas horas de media diarias dormía o duerme? *

Mark only one oval per row.

	6 o menos	7	8	9	10	11	12 o más
Previo al confinamiento	⬭	⬭	⬭	⬭	⬭	⬭	⬭
Durante el confinamiento	⬭	⬭	⬭	⬭	⬭	⬭	⬭

24. En relación al sueño ¿Cómo valoraría la calidad de las horas de sueño? *

Mark only one oval per row.

	1 Muy malo	2	3	4	5	6	7	8	9	1(b
Previo al confinamiento	⬭	⬭	⬭	⬭	⬭	⬭	⬭	⬭	⬭	(
Durante el confinamiento	⬭	⬭	⬭	⬭	⬭	⬭	⬭	⬭	⬭	(

WLEIS-S

Instrucciones: A continuación, encontrará algunas afi rmaciones sobre sus emociones y sentimientos. Lea atentamente cada frase e indique por favor el grado de acuerdo o desacuerdo con
respecto a las mismas durante este periodo de confinamiento del Covid-19.

25. La mayoría de las veces sé distinguir porqué tengo ciertos sentimientos *

Mark only one oval.

	1	2	3	4	5	6	7	
Totalmente desacuerdo	⬭	⬭	⬭	⬭	⬭	⬭	⬭	Totalmente de acuerdo

26. Tengo una buena comprensión de mis propias emociones *

Mark only one oval.

	1	2	3	4	5	6	7	
Totalmente desacuerdo	⬭	⬭	⬭	⬭	⬭	⬭	⬭	Totalmente de acuerdo

27. Realmente comprendo lo que yo siento *

Mark only one oval.

	1	2	3	4	5	6	7	
Totalmente desacuerdo								Totalmente de acuerdo

28. Siempre sé si estoy o no estoy feliz *

Mark only one oval.

	1	2	3	4	5	6	7	
Totalmente desacuerdo								Totalmente de acuerdo

29. Conozco siempre las emociones de mis amigos a través de sus comportamientos *

Mark only one oval.

	1	2	3	4	5	6	7	
Totalmente desacuerdo								Totalmente de acuerdo

30. Soy un buen observador de las emociones de los demás *

Mark only one oval.

	1	2	3	4	5	6	7	
Totalmente desacuerdo								Totalmente de acuerdo

31. Soy sensible a los sentimientos y emociones de los demás *

Mark only one oval.

	1	2	3	4	5	6	7	
Totalmente desacuerdo								Totalmente de acuerdo

32. Tengo una buena comprensión de las emociones de las personas que me rodean *

Mark only one oval.

	1	2	3	4	5	6	7	
Totalmente desacuerdo								Totalmente de acuerdo

33. Siempre me fijo metas y luego intento hacerlo lo mejor para alcanzarlas *

Mark only one oval.

	1	2	3	4	5	6	7	
Totalmente desacuerdo								Totalmente de acuerdo

34. Siempre me digo a mi mismo que soy una persona competente *

Mark only one oval.

	1	2	3	4	5	6	7	
Totalmente desacuerdo								Totalmente de acuerdo

35. Soy una persona auto-motivadora *

Mark only one oval.

	1	2	3	4	5	6	7	
Totalmente desacuerdo								Totalmente de acuerdo

36. Siempre me animo a mi mismo para hacerlo lo mejor que pueda

Mark only one oval.

	1	2	3	4	5	6	7	
Totalmente desacuerdo								Totalmente de acuerdo

37. Soy capaz de controlar mi temperamento y manejar las dificultades de manera racional *

Mark only one oval.

	1	2	3	4	5	6	7	
Totalmente desacuerdo	⬭	⬭	⬭	⬭	⬭	⬭	⬭	Totalmente de acuerdo

38. Soy capaz de controlar mis propias emociones *

Mark only one oval.

	1	2	3	4	5	6	7	
Totalmente desacuerdo	⬭	⬭	⬭	⬭	⬭	⬭	⬭	Totalmente de acuerdo

39. Me puedo calmar fácilmente cuando me siento enfadado *

Mark only one oval.

	1	2	3	4	5	6	7	
Totalmente desacuerdo	⬭	⬭	⬭	⬭	⬭	⬭	⬭	Totalmente de acuerdo

40. Tengo un buen control de mis propias emociones *

Mark only one oval.

	1	2	3	4	5	6	7	
Totalmente desacuerdo	⬭	⬭	⬭	⬭	⬭	⬭	⬭	Totalmente de acuerdo

POMS

Rodea con un círculo, para cada uno de los adjetivos siguientes, el número que mejor indique cómo te has sentido en la última semana.

41. Enérgico/a *

Mark only one oval.

	0	1	2	3	4	
NADA	⬭	⬭	⬭	⬭	⬭	MUCHÍSIMO

42. Considerado/a con los demás *

Mark only one oval.

	0	1	2	3	4	
NADA						MUCHÍSIMO

43. Cansado/a *

Mark only one oval.

	0	1	2	3	4	
NADA						MUCHÍSIMO

44. Vigoroso/a *

Mark only one oval.

	0	1	2	3	4	
NADA						MUCHÍSIMO

45. Triste *

Mark only one oval.

	0	1	2	3	4	
NADA						MUCHÍSIMO

46. Malhumorado/a *

Mark only one oval.

	0	1	2	3	4	
NADA						MUCHÍSIMO

47. Exhausto/a *

Mark only one oval.

	0	1	2	3	4	
NADA						MUCHÍSIMO

48. Amable *

Mark only one oval.

	0	1	2	3	4	
NADA						MUCHÍSIMO

49. Nervioso/a *

Mark only one oval.

	0	1	2	3	4	
NADA						MUCHÍSIMO

50. Lleno/a de energía *

Mark only one oval.

	0	1	2	3	4	
NADA						MUCHÍSIMO

51. Agotado/a *

Mark only one oval.

	0	1	2	3	4	
NADA						MUCHÍSIMO

52. Comprensivo/a *

Mark only one oval.

	0	1	2	3	4	
NADA						MUCHÍSIMO

53. Molesto/a *

Mark only one oval.

	0	1	2	3	4	
NADA						MUCHÍSIMO

54. Agitado/a *

Mark only one oval.

	0	1	2	3	4	
NADA						MUCHÍSIMO

55. Animado/a *

Mark only one oval.

	0	1	2	3	4	
NADA						MUCHÍSIMO

56. Débil *

Mark only one oval.

	0	1	2	3	4	
NADA						MUCHÍSIMO

57. Desesperanzado/a *

Mark only one oval.

	0	1	2	3	4	
NADA						MUCHÍSIMO

58. Irritable *

Mark only one oval.

	0	1	2	3	4	
NADA						MUCHÍSIMO

59. Activo/a *

Mark only one oval.

	0	1	2	3	4	
NADA						MUCHÍSIMO

60. Melancólico/a *

Mark only one oval.

	0	1	2	3	4	
NADA						MUCHÍSIMO

61. Amistoso/a *

Mark only one oval.

	0	1	2	3	4	
NADA						MUCHÍSIMO

62. Enfadado/a *

Mark only one oval.

	0	1	2	3	4	
NADA	◯	◯	◯	◯	◯	MUCHÍSIMO

63. Con los nervios de punta *

Mark only one oval.

	0	1	2	3	4	
NADA	◯	◯	◯	◯	◯	MUCHÍSIMO

64. Solo/a *

Mark only one oval.

	0	1	2	3	4	
NADA	◯	◯	◯	◯	◯	MUCHÍSIMO

65. Fatigado/a *

Mark only one oval.

	0	1	2	3	4	
NADA	◯	◯	◯	◯	◯	MUCHÍSIMO

66. Servicial *

Mark only one oval.

	0	1	2	3	4	
NADA	◯	◯	◯	◯	◯	MUCHÍSIMO

67. Inquieto/a *

Mark only one oval.

	0	1	2	3	4	
NADA	◯	◯	◯	◯	◯	MUCHÍSIMO

68. Infeliz *

Mark only one oval.

	0	1	2	3	4	
NADA	◯	◯	◯	◯	◯	MUCHÍSIMO

69. Resentido/a *

Mark only one oval.

	0	1	2	3	4	
NADA	◯	◯	◯	◯	◯	MUCHÍSIMO

70. Tenso/a *

Mark only one oval.

	0	1	2	3	4	
NADA	◯	◯	◯	◯	◯	MUCHÍSIMO

BRS

Por favor lea las siguientes afirmaciones. A la derecha de cada una encontrará cinco números, que van desde "1" (Totalmente en desacuerdo) a la izquierda hasta "5" (Totalmente de acuerdo) a la derecha. Señale el número que mejor indique tus sentimientos sobre esa afirmación en general, con independencia del confinamiento por el Covid-19.

71. Tiendo a recuperarme rápidamente después de haberlo pasado mal *

Mark only one oval.

	1	2	3	4	5	
Completamente en desacuerdo	◯	◯	◯	◯	◯	Completamente de acuerdo

72. Lo paso mal cuando tengo que enfrentarme a situaciones estresantes *

Mark only one oval.

	1	2	3	4	5	
Completamente en desacuerdo						Completamente de acuerdo

73. No tardo mucho en recuperarme después de una situación estresante *

Mark only one oval.

	1	2	3	4	5	
Completamente en desacuerdo						Completamente de acuerdo

74. Es difícil para mí recuperarme cuando me ocurre algo malo *

Mark only one oval.

	1	2	3	4	5	
Completamente en desacuerdo						Completamente de acuerdo

75. Aunque pase por situaciones difíciles, normalmente no lo paso demasiado mal *

Mark only one oval.

	1	2	3	4	5	
Completamente en desacuerdo						Completamente de acuerdo

76. Suelo tardar mucho tiempo en recuperarme de los contratiempos que me ocurren en mi vida *

Mark only one oval.

	1	2	3	4	5	
Completamente en desacuerdo						Completamente de acuerdo

Sport Motivation Scale-II

Usando la escala a continuación, indique en qué medida cada uno de los siguientes elementos se corresponde con una de las razones por las que actualmente practica su deporte o entrena durante el confinamiento por Covid-19.

77. Porque me sentiría mal conmigo mismo/a si no dedicara tiempo a la práctica de actividad físico-deportiva *

Mark only one oval.

	1	2	3	4	5	6	7	
Completamente en desacuerdo	◯	◯	◯	◯	◯	◯	◯	Completamente de acuerdo

78. Antes participaba y me esforzaba en los entrenamientos pero ahora me pregunto si debo continuar haciéndolo *

Mark only one oval.

	1	2	3	4	5	6	7	
Completamente en desacuerdo	◯	◯	◯	◯	◯	◯	◯	Completamente de acuerdo

79. Porque es muy interesante aprender cómo puedo mejorar *

Mark only one oval.

	1	2	3	4	5	6	7	
Completamente en desacuerdo	◯	◯	◯	◯	◯	◯	◯	Completamente de acuerdo

80. Porque la práctica de actividad físico-deportiva refleja la esencia de quien soy *

Mark only one oval.

	1	2	3	4	5	6	7	
Completamente en desacuerdo	◯	◯	◯	◯	◯	◯	◯	Completamente de acuerdo

81. Porque la gente que me importa se molestaría conmigo sino lo hiciera *

Mark only one oval.

	1	2	3	4	5	6	7	
Completamente en desacuerdo	◯	◯	◯	◯	◯	◯	◯	Completamente de acuerdo

82. Porque creo que es una buena manera de desarrollar aspectos que valoro de mí mismo/a *

Mark only one oval.

	1	2	3	4	5	6	7	
Completamente en desacuerdo	◯	◯	◯	◯	◯	◯	◯	Completamente de acuerdo

83. Porque me sentiría mal si no participara y me esforzara en los entrenamientos *

Mark only one oval.

	1	2	3	4	5	6	7	
Completamente en desacuerdo	◯	◯	◯	◯	◯	◯	◯	Completamente de acuerdo

84. Porque creo que los demás desaprobarían que no lo hiciera *

Mark only one oval.

	1	2	3	4	5	6	7	
Completamente en desacuerdo	◯	◯	◯	◯	◯	◯	◯	Completamente de acuerdo

85. Porque me permite descubrir nuevas actividades físico-deportivas *

Mark only one oval.

	1	2	3	4	5	6	7	
Completamente en desacuerdo	◯	◯	◯	◯	◯	◯	◯	Completamente de acuerdo

86. Tengo la impresión de que no soy capaz de tener éxito en las actividades físico-deportivas que realizo *

Mark only one oval.

	1	2	3	4	5	6	7	
Completamente en desacuerdo	◯	◯	◯	◯	◯	◯	◯	Completamente de acuerdo

87. Porque la práctica de actividad físico-deportiva es parte fundamental de mi vida *

Mark only one oval.

	1	2	3	4	5	6	7	
Completamente en desacuerdo	⬭	⬭	⬭	⬭	⬭	⬭	⬭	Completamente de acuerdo

88. Porque la actividad físico-deportiva es una forma para desarrollarme *

Mark only one oval.

	1	2	3	4	5	6	7	
Completamente en desacuerdo	⬭	⬭	⬭	⬭	⬭	⬭	⬭	Completamente de acuerdo

89. Realmente no me siento capacitado/a para la práctica físico-deportiva *

Mark only one oval.

	1	2	3	4	5	6	7	
Completamente en desacuerdo	⬭	⬭	⬭	⬭	⬭	⬭	⬭	Completamente de acuerdo

90. Porque la práctica actividad físico-deportiva hace que viva según mis principios más profundos *

Mark only one oval.

	1	2	3	4	5	6	7	
Completamente en desacuerdo	⬭	⬭	⬭	⬭	⬭	⬭	⬭	Completamente de acuerdo

91. Porque obtengo recompensa de la gente que me rodea cuando lo hago *

Mark only one oval.

	1	2	3	4	5	6	7	
Completamente en desacuerdo	⬭	⬭	⬭	⬭	⬭	⬭	⬭	Completamente de acuerdo

92. Porque me siento mejor conmigo mismo cuando participo y me esfuerzo en los entrenamientos *

Mark only one oval.

	1	2	3	4	5	6	7	
Completamente en desacuerdo								Completamente de acuerdo

93. Por el placer que siento mientras realizo actividad físico-deportiva *

Mark only one oval.

	1	2	3	4	5	6	7	
Completamente en desacuerdo								Completamente de acuerdo

94. Porque es una de las mejores formas de desarrollar otros aspectos de mí mismo/a *

Mark only one oval.

	1	2	3	4	5	6	7	
Completamente en desacuerdo								Completamente de acuerdo

95. Pulse enviar para finalizar el cuestionario. GRACIAS POR SU PARTICIPACIÓN.

This content is neither created nor endorsed by Google.

Google Forms
